# Availability and Affordability of Oncology Drugs in 2012-2021 in China and the United States

**DOI:** 10.3389/fonc.2022.930846

**Published:** 2022-07-22

**Authors:** Huiyao Huang, Qi Zhu, Man Ga, Dawei Wu, Xinyu Meng, Shuhang Wang, Hong Fang, Yu Tang, Ning Li

**Affiliations:** ^1^ Department of Clinical Trials Center, National Cancer Center/National Clinical Research Center for Cancer/Cancer Hospital, Chinese Academy of Medical Sciences and Peking Union Medical College, Beijing, China; ^2^ School of Basic Medicine and Clinical Pharmacy, China Pharmaceutical University, Nanjing, China; ^3^ Faculty of Medicine, Dentistry and Health Sciences, The University of Melbourne, Melbourne, VIC, Australia

**Keywords:** oncology, anticancer drug, China, USA, availability, affordability

## Abstract

**Objective:**

To systematically summarize the landscape and characteristics of all approved new anticancer drugs for the last 10 years in China and the United States (US) to further inform the trend, current state, and existing gap in the availability and affordability of cancer medicine between the two countries.

**Methods:**

Mainly based on the Pharmcube database, a list and detailed information of anticancer drugs approved in China and the United States were acquired. The annual number, time lag, and basic characteristics, including drug type, mechanism, enterprise type, indication population, drug target, and cancer type of approved drugs were compared.

**Results:**

Eighty-seven and 118 new anticancer drugs were approved in China and the US, respectively, showing a stable trend in the US, while a significant increase was observed after 2016 in China. Of the 42 cancer medicines launched in both countries, the US took precedence, and the median time lag markedly decreased, from 6.53 years in 2012 to 0.88 years in 2020. A total of 14.4% of drugs were applicable to children in the US, while only 2.3% were applicable in China, and there was no difference in drug type and enterprise. Thirty-one and 43 targets were explored, with respect to 27 and 36 cancer types in China and the US, respectively, during the period. In addition, the expenditure of drugs on PD-1 and PD-L1 in China was generally lower than that in America.

**Conclusion:**

The availability of new anticancer drugs has increased dramatically in the past decade, particularly in China. Compared with the US, the launch of new anticancer drugs in China lags behind, but the time lag has been shortened significantly, and better affordability is observed in immune drugs. More attention should be given to differentiated innovation, and unmet medical needs in special populations like childhood tumors, which are important directions of new drug R&D in China.

## Introduction

Cancer is one of the greatest public health challenges globally and is responsible for almost 10 million deaths in the world (1). Additionally, cancer has broad societal impacts beyond the negative effects it has on patients’ health life, such as economic burden and productivity losses for cancer patients and their family caregivers (2, 3). The availability of safe and effective anticancer therapies is a core premise for effective national cancer control plans (4), while the affordability of such anticancer therapies is a critical obstacle to the aspiration of delivering sustainable cancer care (5). Access to cancer medicines has attracted the attention of stakeholders at all levels, from patients and clinicians to governments, deemed as the key to national health service capacity (6).

In fact, to address the unmet needs in a broad cancer population, cancer drug research and development as well as regulatory science have advanced rapidly worldwide in the past decade (7). Expedited programs and adaptive trials have been increasingly and frequently adopted by regulatory agencies (8). However, research on the availability and affordability of antitumor drugs at the national or regional levels is currently deficient (9, 10). China and the US are not only the top two countries in terms of cancer incidence (11, 12) but also representative of developing and developed countries. In view of the importance and with consideration of cancer medicine, this study seeks to systematically summarize the landscape and characteristics of all approved anticancer drugs for the last 10 years in China and the US to further define the trend, current state, and existing gap in the availability and affordability of cancer medicine between the two countries.

## Materials and methods

### Data Source

Information in the study was captured from the following databases. First, based on the Pharmcube database (13), a preliminary list and basic characteristics of anticancer drugs approved to the market were obtained, including drug name, listed country, initial listed date in China and the US, drug type, mechanism, enterprise type, drug target, cancer site, and indication population. Indications were classified according to the tumor site, and multiple indications approved for the same site are not counted repetitively. Cancer sites were further reclassified into rare tumors and childhood tumors, which were two kinds of indication populations with specific concern, according to the standard criterion issued by the National Cancer Center of China (14).

Furthermore, the prices of the PD-1/PD-L1 drugs involved were collected from hybrid sources, including the National Health Care Security Administration, IBM Micromedex Red Book Online (IBM), and the official websites of companies (15). To compare the economic burden of immune checkpoint inhibitors in China and the US, pricing information was shown, including drug name, pharmaceutics, price, specification, dose, frequency, total times, and annual cost. The annual cost of cancer patients with PD-1/PD-L1 was calculated as follows: Annual cost= Unit price per * dose * total times.

### Data Quality Control

The study summarized new anticancer drugs approved in China and the US in the last decade; thus, the following exclusion criteria were formulated uniformly in advance: 1) cancer supportive drugs; 2) generic cancer drugs or cancer biosimilars; 3) anticancer drugs listed neither in China nor the US; 4) approved period was not from January 1, 2012, to December 31, 2021; and 5) withdrawn drugs. To ensure data accuracy and integrity, drug screening and information verification were independently conducted by QZ and MG. If there was any controversy, the third party HYH would be invited for arbitration until consensus was reached.

### Statistical Analysis

The primary endpoints of this study included the number of approved drugs and time lag, which was defined as the drug launch time interval between China and the US. Frequency and percentage were used for the characteristic description of approved drugs, and the chi-square test was used for statistical comparison by drug type, mechanism, enterprise type, and indication population between China and the US. In addition, the whole picture of involved targets and cancer types was depicted numerically. SAS 9.4 was used for data analysis. P<0.05 was considered statistically significant.

## Results

### Availability of Oncology Drugs in China and the US

#### Number of Approved Drugs

**Figure 1:**
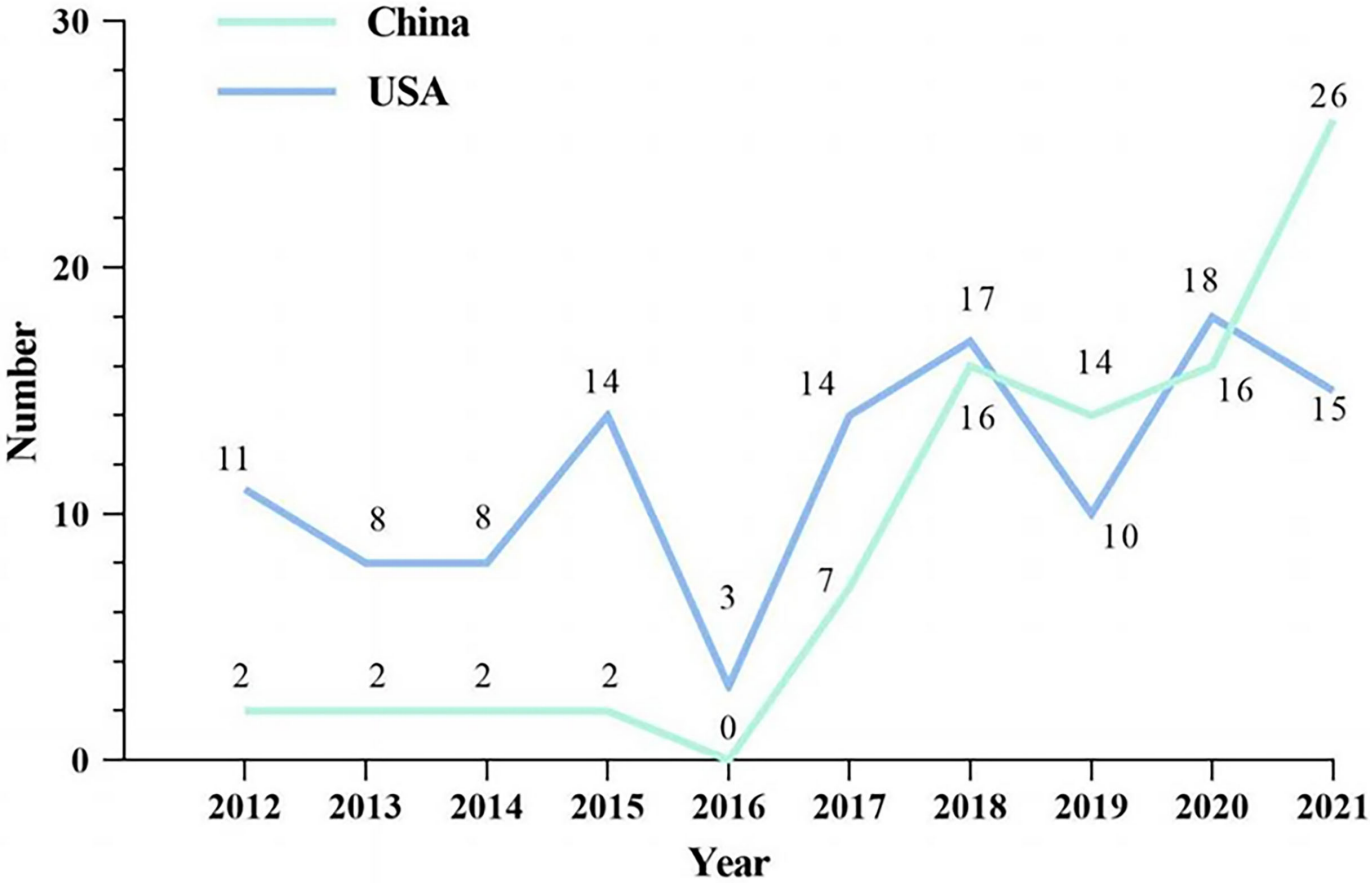
Approved anticancer drugs in China and the USA from 2012 to 2021. Note: The vertical ordinate is the number of approved anticancer drugs. The green line represents the quantitative changes of listed drugs in China while the blue one represents the changes in the USA.

For the last decade, a total of 87 and 118 anticancer drugs have been listed in China and the US, respectively (**Figure 1**). A clear upward trend in approved products of China since 2016 was observed, while the numbers fluctuated yearly in the USA. In 2019, the number of listed drugs in China surpassed that of the US for the first time and reached 26 in 2021.

#### Time Lag of Approved Drugs

**Figure 2:**
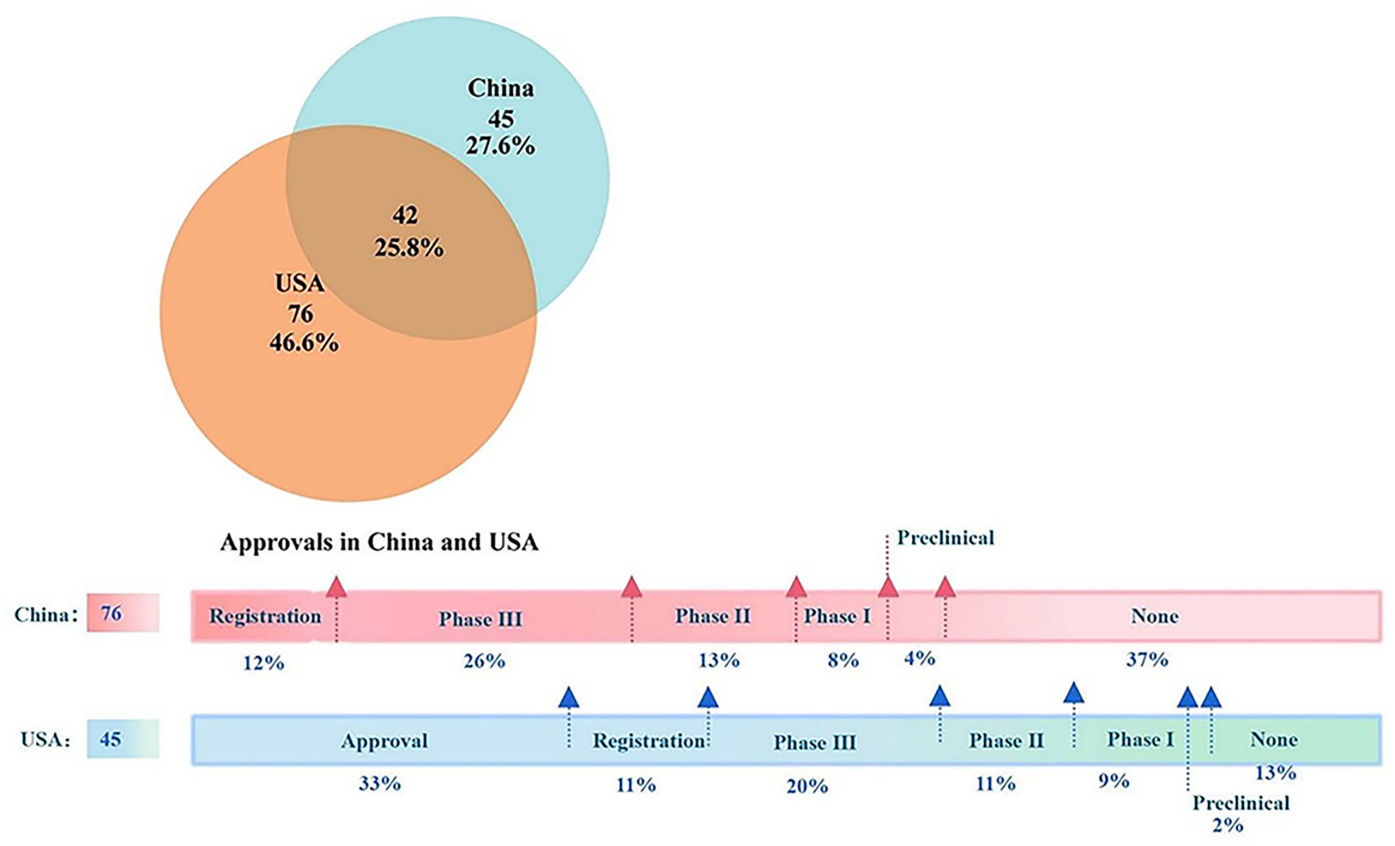
The Venn diagram and the latest progress of listed anticancer drugs in China and the USA. Note: In the upper Venn diagram, the orange circle indicates listed drugs in the USA while the blue one indicates marketed products in China. The overlapping region indicates the products listed in both countries. The horizontal bars underneath described the research progress of drugs that have not been approved in the two countries from 2012 to 2021. Out of 45 anticancer drugs only approved in China in this period, 15 were approved in the United States before 2012.

Among all the approved anticancer drugs during the period, 45 (27.6%) drugs were approved only in China, while 76 (46.6%) drugs were granted only to the US (**Figure 2**). In terms of the development progress of unapproved drugs in both countries between 2012 and 2021, 37% of research drugs in China have not entered the preclinical or clinical trial stage. Only 12% of the drugs were in the registration stage, representing them at the marketing reporting phase. In addition, 8%, 13%, and 26% of the drugs were in clinical phases I, II, and III, respectively. On the other hand, 15 drugs in the US were approved before 2012, which were not reflected in the Venn diagram. Out of the remaining 30 anticancer drugs, only six (13%) had no development reported, 18 (40%) have entered clinical trials, and another 11% were applying for listing. Finally, the study found that China and the US have the fewest drugs in the preclinical stage, accounting for 4% and 2%, respectively.

**Figure 3:**
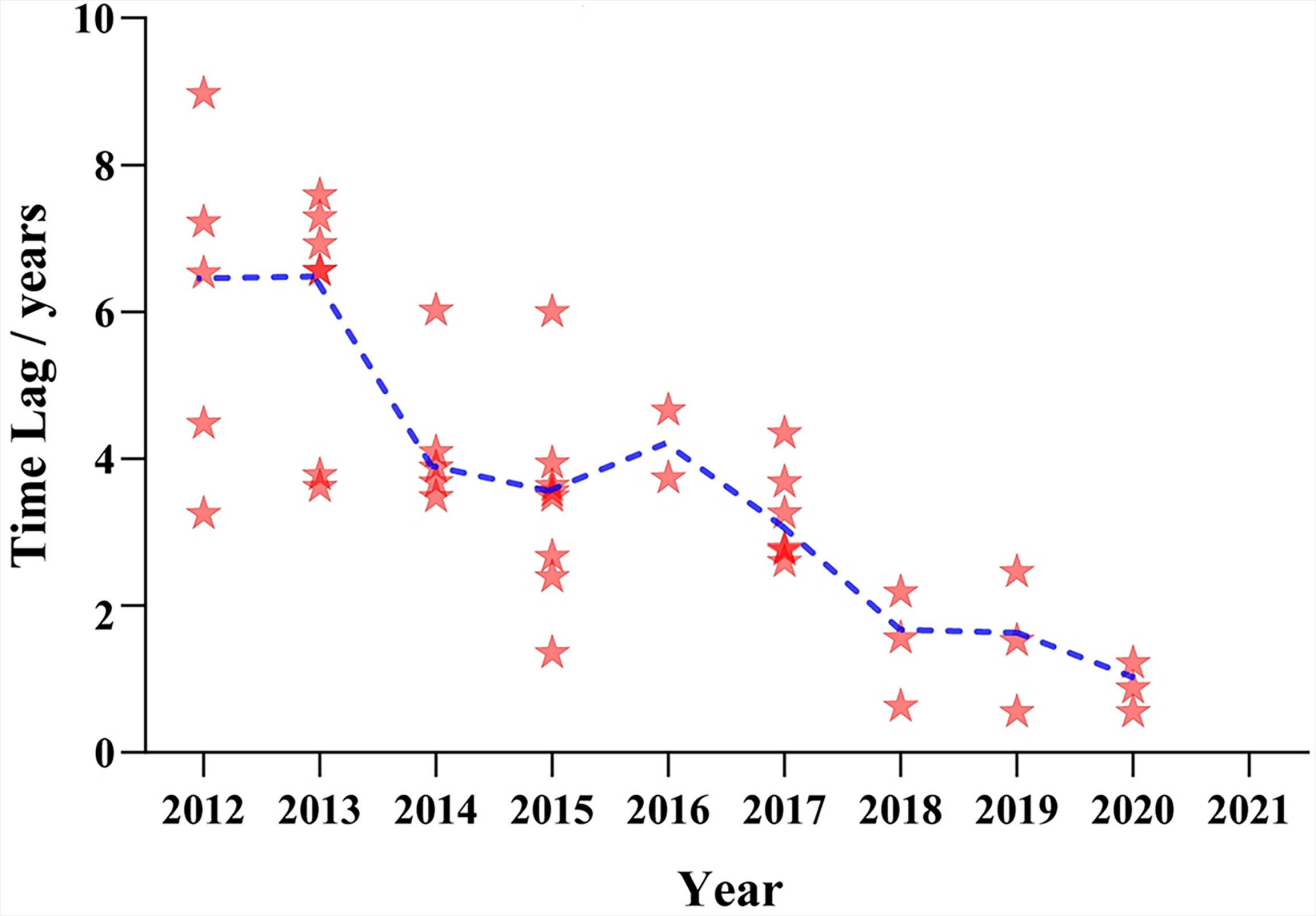
Listing time lag of anticancer drugs between China and the USA from 2012 to 2021. Note: The horizontal axis indicates the approval time of anticancer drugs in the USA, and the vertical axis indicates the approved time lag between China and the USA. Each red star represents one product approved in the two countries. The blue line shows a change tendency of the median time lag from 2012 to 2021.

For the other 42 drugs authorized in both countries, invariably, they all were listed in the US first, with the time lag shorter and shorter year by year. The median time lag ranged from 6.53 years in 2012 to 0.88 years in 2020 (**Figure 3**). In the past 4 years, fewer new drugs approved in the US have been listed in China, with two to three on average. In 2021, new anticancer agents approved by the FDA were not licensed to the Chinese mainland.

### Basic Characteristics of Approved Drugs

Compared with those listed in the US, no significant difference was observed with respect to chemicals (61, 70.1% vs. 82, 69.5%), biologic agents (24, 27.6% vs. 31, 26.3%) and cell therapy (2, 2.3% vs. 5, 4.2%) in China. The two countries had approximate proportions of cytotoxic agents (13, 14.9% vs. 13, 11.0%) and targeted agents (74, 85.1% vs. 105, 89.0%), while China showed more immune agents (13, 14.9% vs. 7, 5.9%, *P*<0.05).

Overall, there seemed to be no difference in the enterprise type between China and the US, but more drugs (57.5%) were developed by global companies in China, which was contrary to the US (47.5%). Regarding the indication population, they had an approximate proportion of drugs on rare tumors (24, 20.3% vs. 22, 25.3%, *P*=0.401), while the US had more drugs approved in childhood tumors (2, 2.3% vs. 17, 14.4%, *P*<0.05). More details are displayed in **Table 1**.

**Table 1:** Characteristics of approved cancer new drugs in China and the USA from 2012 to 2021.

Item		China		USA		*Statistic*		*P-value*
		N	%	N	%			
Drug Type						0.586		0.746
	Chemical	61	70.1%	82	69.5%			
	Biologic agent	24	27.6%	31	26.3%			
	Cell therapy	2	2.3%	5	4.2%			
Mechanism								
	Cytotoxic agent	13	14.9%	13	11.0%	0.697		0.404
	Targeted agent	74	85.1%	105	89.0%	0.697		0.404
	Immune agent	13	14.9%	7	5.9%	4.618		<0.05
Enterprise Type						2.011		0.156
	Global	50	57.5%	56	47.5%			
	Domestic	37	42.5%	62	52.5%			
Rare tumor						0.705		0.401
	Yes	22	25.3%	24	20.3%			
	No	65	74.7%	94	79.7%			
Childhood tumor						8.368		<0.05*
	Yes	2	2.3%	17	14.4%			
	No	85	97.7%	109	92.4%			

^*^Fisher’s exact test.

N, number of the approved drugs in the specific area. %, the proportion of the number of a certain category in all the approved products.

### Target Distribution of Approved Drugs

**Figure 4:**
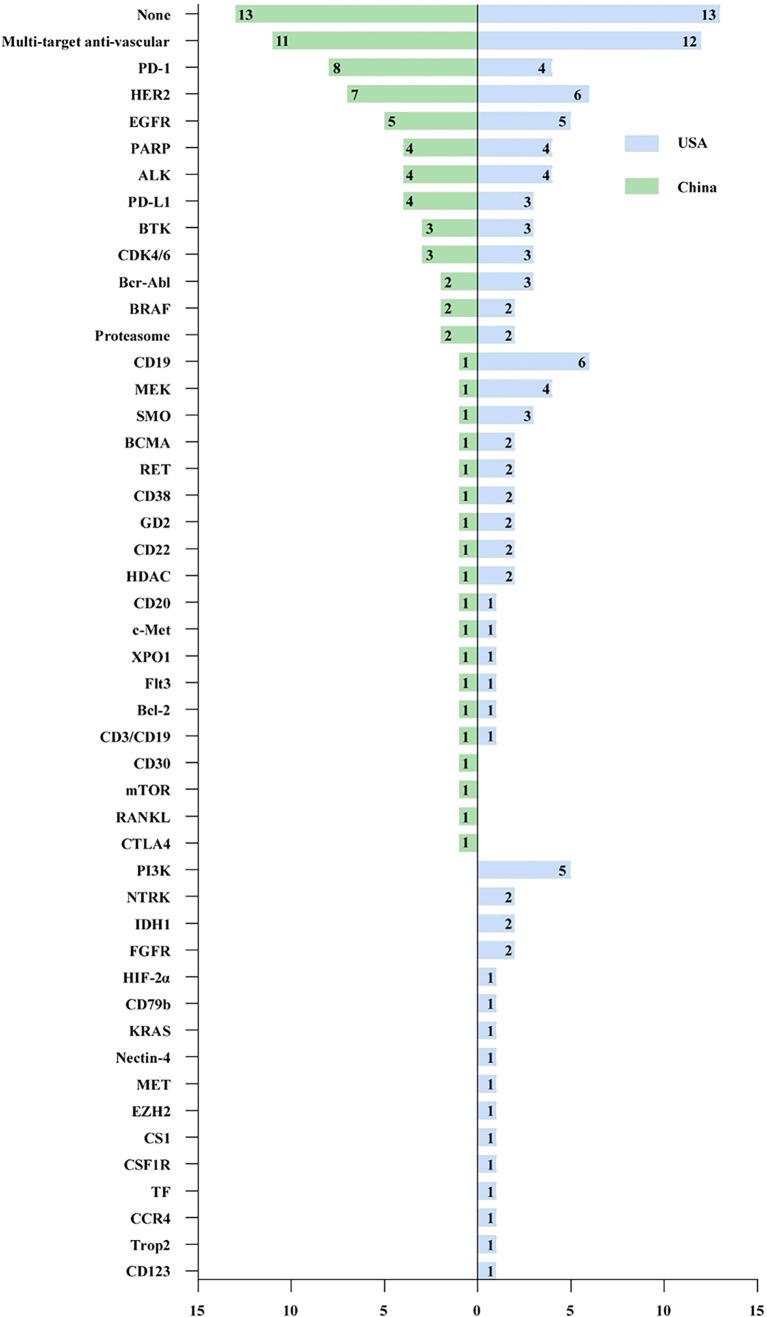
Target distribution of new cancer drugs in China and the USA from 2012 to 2021.

As shown in **Figure 4**, 31 and 43 targets have been explored in China and the US, respectively, in the past decade. In general, there was a large degree of overlap between China and the USA in the selection of targets, among which 27 targets were developed from 2012 to 2021. Multitarget anti-vascular therapy was the most developed target in China and the USA, with 11 (12.6%) and 12 approvals (10.2%), respectively. The three most common targets developed in China were PD-1 (8, 9.2%), HER2 (7, 8.0%), and EGFR (5, 5.7%), which were slightly different from those in America, which were CD19 (6, 5.1%), HER2 (6, 5.1%), and EGFR (5, 4.2%). In addition, four targets were not explored in the US, while 16 targets were in the US for which there were no drugs approved in China.

### Cancer Site of Approved Drugs

**Figure 5:**
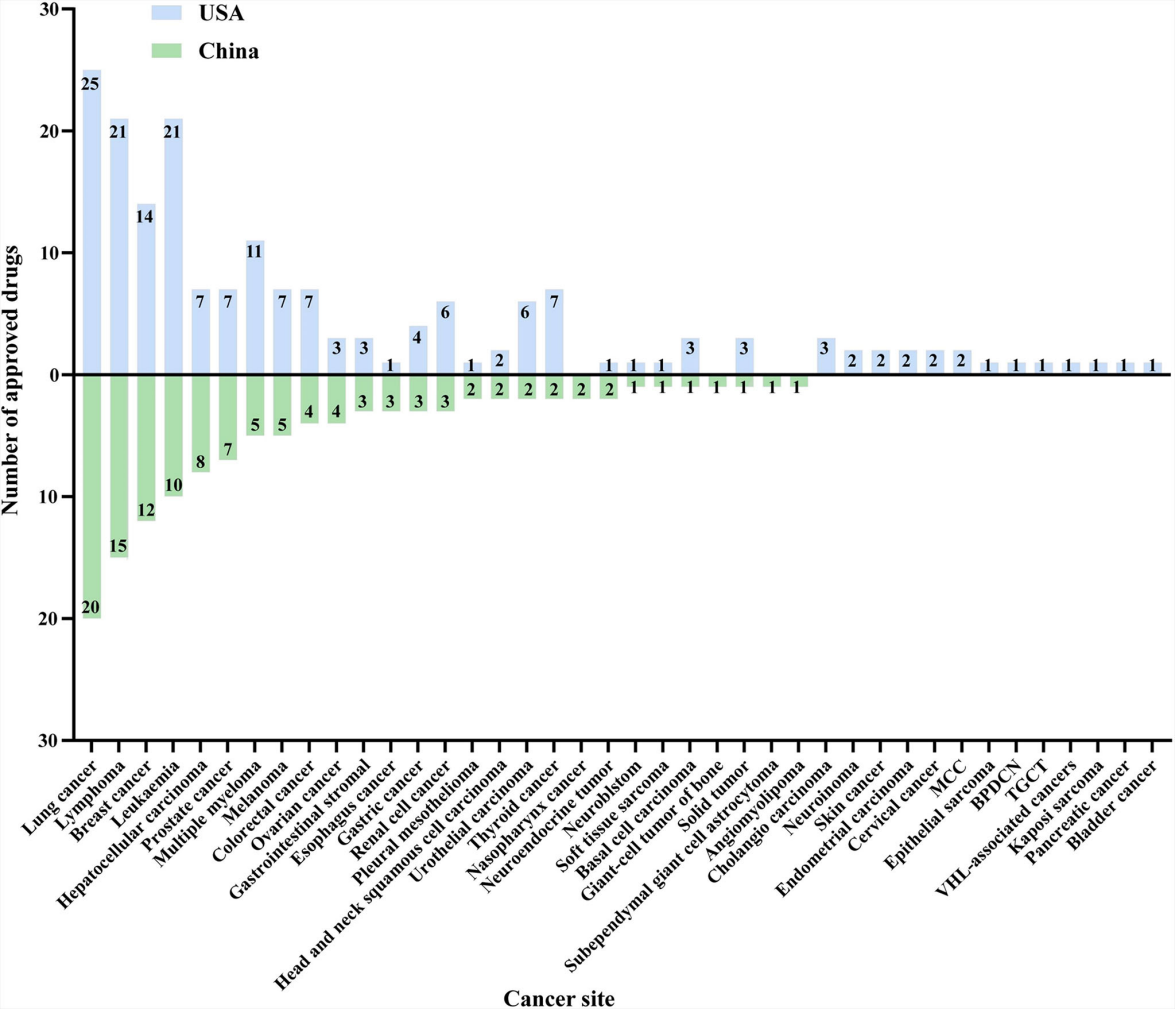
Cancer site distribution of approved new cancer drugs in China and the USA from 2012 to 2021. Note: MCC, Merkel cell carcinoma. BPDCN, blastic plasmacytoid dendritic cell neoplasm. TGCT, tenosynovial giant cell tumor.

All indications for approved new drugs were summarized and categorized by cancer type, which included not only the first indication but also the indications for subsequent supplementary applications. Similar to the targets, there were 27 and 36 cancer types involved in China and the US, respectively. Among the known approved drugs, four cancer types were not involved in the US, and 13 in China had no new approved drugs. The top 10 approved cancer types in China were lung cancer (20, 23.0%), lymphoma (15, 17.2%), breast cancer (12, 13.8%), leukemia (10, 11.5%), hepatocellular carcinoma (8, 9.2%), prostate cancer (7, 8.0%), multiple myeloma (5, 5.7%), melanoma (5, 5.7%), colorectum (4, 4.6%), and ovarian cancer (4, 4.6%), while those approved in the USA were lung cancer (25, 21.2%), lymphoma (21, 17.8%), leukemia (21, 17.8%), breast cancer (14, 11.9%), multiple myeloma (11, 9.3%), hepatocellular carcinoma (7, 5.9%), prostate cancer (7, 5.9%), melanoma (7, 5.9%), colorectum (7, 5.9%), and thyroid cancer (7, 5.9%) (**Figure 5**).

### Price of Approved Immune Checkpoint Inhibitors

This study included information on the prices and annual costs of 11 and seven types of PD-1 and PD-L1 drugs approved in China and the US, respectively (**Table 2**). Compared with the US, the cost of drugs in China was generally lower, including pembrolizumab, nivolumab, and atezolizumab, which in China were almost half the cost than in the US, and the annual cost of zimberelimab was only US$13,000.

**Table 2:** Price information of PD-1 and PD-L1 approved in China and the USA.

Drug Name	Licensee	Price/dollars	Specification/mg	Dose/mg	Frequency	Total times/Year	Annual cost/10,000 dollars
Camrelizumab	Hengrui	China:3114	200	200	q3w	17	China:5.3
toripalimab	Junshi	China:1132	240	240	q2w	26	China:2.9
tislelizumab	BeiGene	China:1681	100	200	q3w	17	China:5.7
Sintilimab	Innovent	China:1233	100	200	q3w	17	China:4.2
Penpulimab	Akeso/Chaitai	China:767	100	200	q2w	26	China:4.0
Zimberelimab	Gloria/Wuxi	China:519	120	120	q2w	26	China:1.3
Envafolimab	Simcere/3D Med	China:940	200	200	qw	52	China:4.9
Pembrolizumab	MSD	China:2818 US:5102	100	200	q3w	17	China:9.6 US:17.3
Nivolumab	BMS	China:1456 US:3697	100	200	q2w	26	China:7.6 US:19.2
Atezolizumab	Genentech.	China:5158 US:9411	1200	1200	q3w	17	China:8.8 US:16.0
Durvalumab	AstraZeneca	China:2844 US:3697	500	500	q2w	26	China:7.4 US:9.6
Avelumab	Emd Serono	US:1692	200	600	q2w	26	US:13.2
Cemiplimab	Regeneron	US:9362	350	350	q3w	17	US:15.9
Dostarlimab	GSK	US:10304	500	500	q3w	17	US:17.5

## Discussion

This study explored the systematic summary and comparison of listed anticancer drugs in China and the United States in the last 10 years. The results showed fundamental progress in the availability of anticancer drugs over the past decade in both countries, with a total of 205 drugs approved, which is also a direct response to the growing number of related clinical trials globally (16, 17).

In terms of the annual growth of listed drugs, the development of anticancer drugs in China has been on a fast track since 2016; 90.8% of listed drugs in China were approved after 2015, and the drug lag was significantly reduced year by year. This is largely associated with continuous R&D investment by biopharmaceutical companies and indelible efforts of the government. To overcome the drug lag and cultivate a more innovation-friendly drug R&D ecosystem in China, the NMPA has implemented priority examination and approval since 2015, emphasizing clinical value-oriented approval (18). The milestone policy “Opinions on Reforming the Review and Approval System of Drugs and Medical Devices” was also issued to improve clinical development efficiency in China, together with many compatible regulatory reforms, such as restrictions on imported drug approval, which loosened that clinical trial data acquired abroad were accepted (11, 19).

In 2020, a series of expedited programs were launched and updated to accelerate drug development and review in China, including breakthrough therapy, conditional approval, and priority review, which caused an increase in annual approvals (19). In contrast, the innovation system of R&D in the US has long been established, including expedited programs of drug reviews, which is why annual approvals were relatively stable (20).

Incentivized by accelerated policies and fast-track modes, nearly 60% of drugs in China have been in the clinical and marketing filing stage in terms of the progress of new drug development, despite the gap with the US. Different from 2018-2020, 77% of drugs (20/26) listed in China in 2021 were from domestic enterprises, which was related to the transformation of traditional pharmaceutical companies and the emergence of new biotech companies.

Based on the fast follow-up model, the development of new drug targets in China has achieved remarkable results. In general, the target of exploration in the USA was more extensive, basically fully covering the R&D pipeline in China, involving several emerging targets and undruggable targets, such as HIF-α2, CCR4, and KRAS G12C (21–23). In addition, drugs targeting CD30, mTOR, RANKL, and CTLA-4 were approved by the FDA before 2012. It was further demonstrated that the excavation of new targets in China was insufficient, which was similar to previous research results based on clinical trial layouts (24).

Actually, domestic enterprises were very active in the development of immune checkpoint inhibitors in China (25). Within 4 years, eight PD-1 and four PD-L1 medications were approved by NMPA. It is undeniable that the pattern of fast follow-up met the needs of Chinese patients for accessible and high-quality tumor therapy drugs to some extent but also helped innovative Chinese companies to enter the international stage. However, repeated development may occupy innovative resources and hinder the development of emerging targets (26). In addition, the dilemma of sustainable development of enterprises caused by homogeneous competition represented by PD-1 has become a new challenge (27, 28). Since 2020, PD-1 pharmaceutical companies represented by Innovent and Junshi have gone overseas to seek globalization routes, suggesting that returning to value-oriented original research and innovation is the most fundamental way.

The expenditure on anticancer drugs has always been the most concerning issue in China, and its affordability directly affects the sustainable development, quality of life, and lifespan of patients (29). Therefore, China has repeatedly negotiated medical insurance to reduce drug costs. Without considering medical insurance and various preferential policies, the cost of immune checkpoint inhibitors on the market has formed three levels: the treatment cost in the US was the highest, the drugs imported from the US to China were second, and the domestic drugs in China were the lowest. Under the national medical insurance negotiation at the end of 2021, the annual cost of the four approved PD-1s in China that entered medical insurance fell below $60,000 USD. In addition, other imported drugs basically have patient assistance programs. In the US, despite the high cost of treatment, abundant and diverse medical insurance policies there will give strong support to improve drugs’ affordability. Different PD-1s also have diverse Patient Assistance Programs, such as nivolumab, including the Good Days, PAN Foundation, and BMS3 Assist Program, either with financial assistance or with help under federal programs and commercial insurance. Generally, China and the US have made good progress on the way to universal health coverage (UHC) but remain facing the long-standing challenges related to accessibility, affordability, and quality of services (29–31). Although medical insurance negotiations have significantly improved access to drugs, the sustainable innovative ecosystem for drug development has suffered. Centering on the concept of differentiated clinical value, improving the commercial insurance and price payment system is critical for Chinese pharmaceutical companies and regulatory agencies.

On the whole, the products approved to the market in the past 10 years were dominated by common cancer types such as lung and breast cancer. In the development of typical tumors, China still faces a large number of unmet clinical needs (31). For instance, three new drugs have been approved for gastric cancer and oesophageal cancer, and medicines with high mortality represented by pancreatic cancer are even deficient. The study also shows that more drugs for childhood tumors (17 vs. 2) have been approved in the US than in China, which is a direct response to the known R&D strategy of choosing unmet clinical needs as the first indication to achieve the fastest market in the US (28). With the establishment of expedited programs in China in 2020, we believe that more R&D will be laid out on childhood tumors in China under the guidance of patient-centralized and value-based development (8, 18).

This is the first comparative study on the availability and affordability of anticancer new drugs in China and the US. There are some limitations in this study. Firstly, due to data availability, only the first indication of approved drugs and drug price of PD-1 and PD-L1 were collected and compared, which could reflect the between-country gap in research orientation and cancer drug affordability to a limited extent. Statistics on the time lag of new drug approval between the two countries were constrained to the 42 drugs approved within the study period; actually, there were another 15 drugs listed in both countries but approved before 2012 in the US. Last but not least, drug accessibility is the fundamental indicator that can truly reflect whether patients can receive effective and affordable drugs, which is not studied in this study due to a lack of data sources.

## Conclusion

The availability of new anticancer drugs has increased dramatically in the past decade, particularly in China, which is partially associated with the development strategy of fast-follow and the introduction of expedited programs. The launch of new anticancer drugs in China lags behind, but the between-country time lag has been shortened significantly. Relatively, cancer drug affordability in China is better than that of the US, according to the price of PD-1 and PD-L1 medications. In the future, more attention should be given to differentiated innovation, and unmet medical needs in special populations like childhood tumors, which are important directions of new drug R&D in China.

## Data Availability Statement

The original contributions presented in the study are included in the article/supplementary material, further inquiries can be directed to the corresponding author/s.

## Author Contributions

HH, QZ, and MG contributed to framework planning and draft writing, as well as data quality control, analysis, and interpretation. DW, XM, SW, HF, and YT provided data and technical support, participated in data quality control, chart production, and data interpretation. NL participated in framework planning and contributed to data interpretation. All the authors reviewed and revised the manuscript.

## Funding

The analysis and interpretation of the manuscript were supported by the Chinese Academy of Medical Sciences Innovation Fund for Medical Sciences (Construction and Application of Clinical Trial and Institution Evaluation System (2021-I2 M-1-045) and Beijing Municipal Health Commission, Beijing Demonstration Research Ward (BCRW20200303).

## Conflict of Interest

The authors declare that the research was conducted in the absence of any commercial or financial relationships that could be construed as a potential conflict of interest.

## Publisher’s Note

All claims expressed in this article are solely those of the authors and do not necessarily represent those of their affiliated organizations, or those of the publisher, the editors and the reviewers. Any product that may be evaluated in this article, or claim that may be made by its manufacturer, is not guaranteed or endorsed by the publisher.
